# Management of Extensively Drug-Resistant Tuberculosis in Peru: Cure Is Possible

**DOI:** 10.1371/journal.pone.0002957

**Published:** 2008-08-13

**Authors:** Cesar A. Bonilla, Aldo Crossa, Hector O. Jave, Carole D. Mitnick, Ronal B. Jamanca, Cesar Herrera, Luis Asencios, Alberto Mendoza, Jaime Bayona, Matteo Zignol, Ernesto Jaramillo

**Affiliations:** 1 Ministry of Health, Lima, Peru; 2 Socios en Salud Sucursal Peru, Lima, Peru; 3 Partners In Health, Boston, Massachusetts, United States of America; 4 Harvard Medical School, Department of Social Medicine, Boston, Massachusetts, United States of America; 5 Nacional Institute of Health, Lima, Peru; 6 World Health Organization, Geneva, Switzerland; University of Stellenbosch, South Africa

## Abstract

**Aim:**

To describe the incidence of extensive drug-resistant tuberculosis (XDR-TB) reported in the Peruvian National multidrug-resistant tuberculosis (MDR-TB) registry over a period of more than ten years and present the treatment outcomes for a cohort of these patients.

**Methods:**

From the Peruvian MDR-TB registry we extracted all entries that were approved for second-line anti-TB treatment between January 1997 and June of 2007 and that had Drug Susceptibility Test (DST) results indicating resistance to both rifampicin and isoniazid (i.e. MDR-TB) in addition to results for at least one fluoroquinolone and one second-line injectable (amikacin, capreomycin and kanamycin).

**Results:**

Of 1,989 confirmed MDR-TB cases with second-line DSTs, 119(6.0%) XDR-TB cases were detected between January 1997 and June of 2007. Lima and its metropolitan area account for 91% of cases, a distribution statistically similar to that of MDR-TB. A total of 43 XDR-TB cases were included in the cohort analysis, 37 of them received ITR. Of these, 17(46%) were cured, 8(22%) died and 11(30%) either failed or defaulted treatment. Of the 14 XDR-TB patients diagnosed as such before ITR treatment initiation, 10 (71%) were cured and the median conversion time was 2 months.

**Conclusion:**

In the Peruvian context, with long experience in treating MDR-TB and low HIV burden, although the overall cure rate was poor, a large proportion of XDR-TB patients can be cured if DST to second-line drugs is performed early and treatment is delivered according to the WHO Guidelines.

## Introduction

Extensively drug-resistant tuberculosis (XDR-TB) is defined as a disease caused by *Mycobacterium tuberculosis* isolates resistant to at least isoniazid and rifampicin (which is the definition of multidrug-resistant tuberculosis, MDR-TB) plus to at least one fluoroquinolone and one second-line injectable (capreomycin, kanamycin or amikacin) [1]. XDR-TB was first introduced as a term in the medical literature in 2006 by a survey of second-line anti-TB drug resistance among MDR-TB isolates collected worldwide [2]. The same year, the international community was alarmed by the report of an outbreak of XDR-TB in the South African province of KwaZulu Natal associated to 98% lethality and short survival time [3]. Since then, XDR-TB has been reported by several studies [4], [5], [6], [7] and detected in 46 countries worldwide [8]

Diagnosing XDR-TB is very challenging as it requires capacity to perform Drug Susceptibility Tests (DSTs) for fluoroquinolones and second-line injectables in addition to first-line anti-TB drugs. In Peru, such testing has been performed since 2005 at the National Reference Laboratory of the Instituto Nacional de Salud (INS). Earlier, DSTs for first-line anti-TB drugs were done nationwide in a quality-assured manner, and second-line anti-TB drugs could only be tested in limited quantity at the Massachusetts State Laboratory Institute (MSLI) in the United States, as part of an international collaboration to implement the so-called DOTS-Plus project [9], [10], [11].

In addition to diagnosis, treatment for XDR-TB presents an even larger challenge due to the limitations in designing treatment regimens. Treatment outcomes of XDR-TB patients vary between different programmes and regions of the world with 30% cure rates among the XDR-TB cases diagnosed in Latvia between 2000 and 2002 [1] compared to 98% mortality among those reported in the KwaZulu Natal outbreak [3]. The very high HIV prevalence in this area of the world [12] could explain the high mortality as HIV is known to worsen TB and MDR-TB treatment outcomes [13].

The burden of XDR-TB and its treatment outcomes have not yet been reported for Peru, a country where second-line anti-TB drugs have been administered (via standardized, empiric or individualized treatment regimens) through the National TB control Programme (NTP) for more than ten years [9]. Like Latvia, Peru has a relatively low burden of HIV compared to South Africa [12] and thus provides a different public health context for the emergence of XDR-TB.

The aim of this study is to contribute to the growing knowledge on XDR-TB epidemiology and management by describing the incidence of XDR-TB cases reported in the Peruvian National MDR-TB registry over a period of more than ten years (January 1997 to June of 2007) and to present the treatment outcomes for a cohort of these patients.

## Methods

### Study population

The selection criteria for which patients receive treatment with second-line drugs through the NTP in Peru has been changing over the last decade [9] and have been recently standardized in the 2005 NTP guidelines [11]. At the moment patients suspected of having MDR-TB are evaluated by either the national or the regional expert committees (CER) to be approved for second-line anti-TB treatment if they fulfil any of the following criteria: persistent culture-positive results at the fourth month of therapy with first-line drugs; documented household contact of a confirmed MDR-TB case; or MDR-TB diagnosed through DST. For cases waiting for DST results, these committees approve standardized or empirical treatment regimens (STR and ETR respectively), depending on whether the resistance pattern of the source case is known. (STR is composed of: 4KCxEtEZ/14CxEtEZ where K = kanamycin, Cx = ciprofloxacin, Et = ethionamide, E = ethambutol, Z = pyrazinamide). A sputum sample is then collected for smear, culture and DST. Regional laboratories perform initial first-line DSTs and, as of March of 2005, culture positive samples are sent to the INS where DSTs are performed for first and second-line drugs. DST results are used by the CER to tailor the individualized treatment regimen (ITR) [11], [14]. Prior to the publication of the Peruvian official treatment guidelines for MDR-TB in 2005 [15], second-line DST results were done through the MSLI, though this was not the general practice.

The CER tailors ITR for MDR-TB and XDR-TB cases generally based on the available DST results. Regimens consist of at least five different drugs to which the patient is susceptible, with first line drugs utilized when possible. Cases that are suspected of, or the DST shows resistance to ciprofloxacin are generally given moxifloxacin (in rare occasion, gatifloxacin was used). Injectable drugs are administered daily and indicated in order of efficacy depending, on resistance: streptomycin, kanamycin and capreomycin. Second line bacteriostatics (ethionamide, cicloserine and *P*-aminosalicylic acid) are then added to complete the regimen. Further details on treatment regimens and the clinical care practices used for MDR-TB patients are described in detail elsewhere [16], [17].

All patients approved for treatment with second-line anti-TB drugs through the NTP are routinely recorded in a National MDR-TB Registry.

For the purpose of this study we extracted all entries in the registry dated between January1997 and June of 2007 and that had DST results indicating MDR-TB. From this group of confirmed MDR-TB cases, we selected all cases that had a DST performed for at least one fluoroquinolone and one second-line injectable at any moment before or during treatment.

### Cohort

For the outcome and culture conversion analyses, we considered the cohort of DST-confirmed MDR patients that began an ITR before March of 2005, when the new national guidelines for MDR-TB treatment [15] where published. We included only patients that had DST results for at least rifampicin, isoniazid, one fluoroquinolone and one second-line injectable (hence being able to confirm or reject XDR-TB diagnosis)[18].

### Definitions and statistical analysis

Internationally recommended definitions of treatment outcomes were used for the analysis [19]. In the case of multiple outcomes, we choose the first outcome assigned to the patient (as indicated by the World Health Organization (WHO) [20]). Similarly, to define culture conversion we used the consensus definition of two consecutive negative cultures collected at least 30 days apart [19], [21]. A positive culture after conversion was considered reversion [21]. In this study, we focused on “final conversion”, or the point at which the culture becomes negative and does not show a positive result again.

XDR-TB cases were compared to the MDR-TB cases who were also confirmed as not having XDR-TB (“MDR group”), meaning that they were shown to not be resistant to any fluoroquinolone or second-line injectable.

In an attempt to capture differences that may result from having more information on resistance before or after starting treatment, both groups (MDR and XDR-TB) were stratified according to the date of the DST that confirmed or excluded XDR-TB diagnosis. Detailed description of the composition of the strata is presented in Table 1.

**Table 1:** Stratification of cases according to the date of the DST and the detection of XDR-TB.

	MDR-TB	XDR-TB
**Stratum 1**	Lab confirmed MDR-TB with 2^nd^-line DSTs for at least a FQ and an INJ, dated BEFORE or up to 31 days after treatment initiation.	Lab-confirmed XDR-TB cases, from DSTs dated BEFORE or up to 31 days after treatment initiation.
**Stratum 2**	Lab confirmed MDR-TB with 2^nd^-line DSTs for at least a FQ and an INJ, dated >31 days AFTER treatment initiation.	Lab-confirmed XDR-TB cases from DSTs dated >31 days AFTER treatment initiation, *without* previous DSTs that include 1 FQ and 1 INJ
**Stratum 3** 1		Lab-confirmed XDR-TB cases from DSTs dated >31 days after treatment, *with* previous DSTs that include 1 FQ and 1 INJ, but hadn't shown to be XDR

1 Patients diagnosed with XDR-TB later than 31 days after treatment initiation are subdivided into strata 2 and 3 to differentiate patients with documented amplification of resistance (strata 3) to patients without enough DST information to document amplification towards XDR-TB, or XDR-TB at the start of treatment.

Comparisons of outcomes between XDR-TB and MDR-TB, and between the different strata was done using Pearson's Chi-squared tests. Dichotomous outcomes and associated odds ratios were computed using Fisher's exact method. Culture conversion was described using Kaplan-Meier graphs and compared with the Log-Rank Test. When constructing conversion curves, individuals were censored if a treatment outcome occurred before conversion, or if conversion did not occur before the end of the 30-month follow-up period, thus cases that default from treatment were censored only if they defaulted before 30 months of treatment. In the analysis of treatment times, Kaplan-Meier curves were also used and considered as censored all cases that did not cure at the end of treatment. Power and sample size calculations for Fisher's exact tests were computed through simulations and reported as estimates with associated confidence intervals. Data analyses were done using SAS version 9.0 (The SAS Institute, North Carolina, USA), and all simulations to estimate power of tests were performed in R version 2.5.1 (Free Software Foundation Inc., Massachusetts, USA). All p-values reported here are two-sided, and significance levels were set to 0.05.

## Results

A total of 7,191 cases were approved for second-line treatment between January 1997 and June of 2007. Among them, 5,335 had a diagnosis of MDR-TB confirmed through DST. Of these, 1,989 (37.3%) also had DST results for at least one fluoroquinolone and one second-line injectable (second-line DST). A total of 119 XDR-TB cases were recorded in the National MDR-TB registry in the study period, which represent 2.2% of all DST-confirmed MDR-TB cases and 6·0% of the MDR-TB cases who had DST results for at least one fluoroquinolone and one second-line injectable in addition to first-line drugs. Forty-two of the 119 (35.3%) XDR-TB cases were tested for HIV and no cases of XDR-TB/HIV co-infection were detected.

The distribution of XDR-TB between sex and age groups did not differ significantly from that of MDR-TB (Table 2). Over 90% of XDR-TB cases were found to live in Lima and its metropolitan area, which follows the distribution of MDR-TB in the country (P = 0.2998).

**Table 2:** Comparison of age, sex and geographic distribution between XDR and MDR.

	XDR	MDR	P-value
	(n = 119)	(n = 1870)	
**Sex**			
***Male***	58.8%(70)	59.7%(1116)	0.7727
***Female***	41.2%(49)	40.3%(754)	
**Age**			
**Median**	27.0	27.0	0.3126
**Range**	(10–78)	(0–82)	
**Geographic Distn.**			
***Lima-Callao***	90.8%(108)	87.5%(1637)	0.2998
***Province***	9.2%(11)	12.5%(233)	

### History of previous anti-TB treatment

Results of the analyses of history of previous anti-TB treatment are shown in Table 3. Cases with XDR-TB had a greater number of previous treatments than those with MDR-TB (2.0 and 1.6 respectively, *P* = 0.0026). Specifically, the XDR group had a significantly higher proportion of patients that underwent two or more previous treatments compared to the MDR group. Among XDR-TB cases 11.5% (95% C.I. = 5.6–17.4) had never been treated before, similarly to what found in the MDR group (11.1%, *95*% *C.I.* = (9.6–12.5)) (*P* = 0.8774). The proportion of XDR-TB cases never treated with second-line anti-TB drugs (40.5%, 95% C.I = 31.4–49.7) was significantly lower than in the MDR group (63.3%, 95 % C.I = (61.1–65.6)) (*P*<0.0001).

**Table 3:** History of prior treatments and number of never treated cases for XDR-TB compared to MDR-TB.

	XDR-TB	MDR-TB	P-value
**Pervious treatments (1^st^+2^nd^ line)**	N = 113	N = 1755	
**Never treated (NT)**	12%(13)	11%(194)	
**1 treatment**	24%(27)	40%(693)	
**2 treatments**	33%(37)	29%(503)	0.0039
**>2 treatments**	32%(36)	21/%(365)	
**NT with first-line regimen**	15%(17)	14%(241)*	0.7115
**NT with second-line regimen**	41%(45)†	63%(1061)‡	<0.0001

* N = 1746.

† N = 111.

‡ N = 1676.

### Treatment outcome analysis

Five of the 119 XDR-TB cases (4.2%) and 40 of the 1,870 MDR-TB cases (2.1%) were excluded from the cohort group used for the outcome analysis because the second-line DST results were dated more than two months after treatment completion. Sixty-one XDR-TB and 732 MDR-TB cases were approved for second-line treatment before March of 2005 (the cut-off date). One XDR-TB patient (1.6%) died before starting treatment, while 22 patients with MDR-TB (2.9%) were approved for second-line treatment but did not start, eight due to death. Four and 24 XDR-TB and MDR-TB patients, respectively, were excluded because they started treatment after the cut-off date. Finally, 38 of the remaining 686 MDR-TB patients and 13 of the 56 XDR cases were excluded from the outcomes analysis because they were still in treatment. Therefore the cohort analyzed for treatment outcomes consisted of 43 XDR-TB cases (37 on ITR and 6 on STR), and 648 MDR-TB cases (494 on ITR and 154 on STR).

Table 4 shows the treatment outcomes of XDR-TB and MDR-TB patients. Overall, 42% of XDR-TB cases were cured. Treatment outcomes were significantly different between patients who underwent STR and ITR (P = 0.0380). Though the proportion of cured patients was higher among those who underwent ITR, the difference was not statistically significant (P = 0.3747).

**Table 4:** Outcome results for all XDR cases by treatment regimen.

	N	Cure (%)	Treatment Completed (%)	Death (%)	Failed (%)	Default (%)	P-value (%)
**XDR**							
**STR**	6	1(17)	2(33)	0(0)	1(17)	2(33)	
**ITR**	37	17(46)	1(3)	8(22)	5(14)	6(16)	
**Total**	43	18(42)	3(7)	8(19)	6(14)	8(19)	0.0380
**XDR vs. MDR (ITR Only)**							
**XDR**	37	17(46)	1(3)	8(22)	5(14)	6(16)	
**MDR**	494	342(69)	30(6)	39(8)	50(10)	33(7)	0.0044
**Stratum 1**							
**XDR**	14	10(71)	1(7)	1(7)	1(7)	1(7)	
**MDR**	334	241(72)	23(7)	26(8)	22(7)	22(7)	0.9999
**Stratum 2**							
**XDR**	16	6(38)	0(0)	5(31)	2(13)	3(19)	
**MDR**	160	101(63)	7(4)	13(9)	28(17)	11(11)	
**Stratum 3**							
**XDR**	7	1(14)	0(0)	2(29)	2(29)	2(29)	0.0130

Comparison of outcomes for XDR vs. MDR patients *under the Individualized Treatment Regimen (ITR)*, stratified according to timing of Drug Sensitivity Test (DST). Includes cases who finished treatment; 13/56 (23%) XDR and 30/524 (5.7%) MDR were still on treatment.

Stratum 1: 2nd-line DST result dated prior to, or up to 31 days after treatment initiation; Stratum 2: 2nd-line DST result dated more than 31 days after treatment initiation; Stratum 3 (only XDR cases): 2nd-line DST result diagnosing XDR-TB, dated more than 31 days after with previous DSTs not having XDR-TB resistance pattern.

The cure rate of XDR-TB patients who underwent ITR was 45.9% compared to 69.2% of those with MDR-TB (*OR* = 0.38, *95% C.I.* = 0.18–0.78). Treatment failures and deaths were significantly higher in the XDR group (OR = 1.38, 95% C.I = 0.51–3.72 and *OR* = 3.2, *95% C.I.* = 1.2–7.8, respectively).

Distribution of outcomes was not significantly different (*P* = 0.9999) between MDR-TB and XDR-TB patients who underwent ITR with a second-line DST dated before or up to 31 days after treatment initiation (stratum 1). In this stratum, 71.4% (10/14) of XDR-TB cases were cured, compared to 68.9% of those with MDR-TB (241/334; *OR* = 0.96, *95% C.I.* = 0.30–3.15). In the same stratum, the proportion of patients who failed or died was statistically similar for MDR and XDR groups (*P* = 0.3888 and 0.3873 respectively).

Table 4 shows that the distribution of outcomes for the XDR group was not significantly different between strata 2 and 3 (*P* = 0.6121). When comparing these two groups to the second strata of MDR-TB patients, only stratum 3 showed a significantly lower percentage of cured XDR-TB cases compared to MDR-TB (*OR* = 0.10, *95% C.I.* = 0.01–0.83). The percentage of cured patients in stratum 2 of XDR-TB cases was 37.5% (6/16), which is not significantly lower than that found in the MDR-TB group (*OR* = 0.35; *95% C.I.* = 0.12–1.01).

### Duration of treatment

In the cohort of patients under ITR, XDR-TB patients received treatment for a significantly longer duration. The median time to cure for XDR-TB patients was 43.1 months, compared to the average 28.5 months that MDR-TB cases spent in treatment (P<0.0005).

For stratum 1, XDR-TB cases were cured in a median of 27.7 months compared to 24.9 months for MDR-TB (P = 0.1790). In the higher strata, MDR-TB patients were cured on average 2 months earlier than the XDR-TB group (P = 0.0063).

### Culture Conversion analysis

Overall, culture conversion in the MDR group occurred at a median of three months compared to that of 26 months in the XDR-TB cohort (P = 0.0006). The stratified analysis suggests that the date of the DST is also associated with culture conversion. Figure 1 shows the Kaplan-Meier graph of the probability of conversion by month of treatment.

**Figure:**
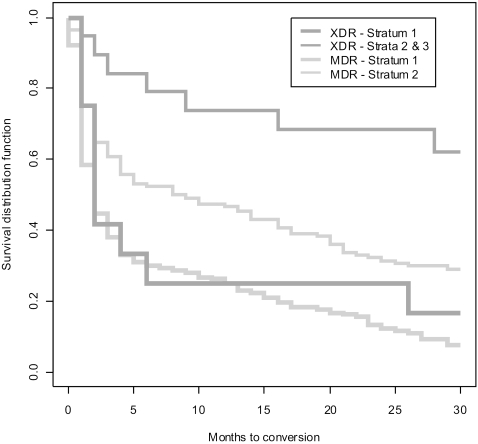


As shown in Figure 1, culture conversion was found to follow a similar path for MDR-TB and XDR-TB cases in stratum 1, with half of the cases reaching culture conversion by the second month in treatment and roughly 75% by the sixth month (*P*<0.3885). Culture conversion was significantly slower in the higher strata, though the difference was more pronounced in the XDR group. Among patients with DST dated more than 31 days after starting treatment (strata 2 and 3), culture conversion occurred significantly quicker in the MDR group (*P* = 0.0220). For both XDR strata with DST after treatment initiation the proportion of patients reaching culture conversion in the first 30 months of treatment was below 50%.

## Discussion

Patients with XDR-TB represent 6.0% of all laboratory-confirmed MDR-TB cases recorded in the Peruvian MDR-TB registry between January 1997 and June of 2007 who had DST results for first-line drugs and for at least one fluoroquinolone and one second-line injectable. In contrast to findings from Latvia where XDR-TB cases are spread countrywide [2], the vast majority of cases in Peru have been found in Lima and its surrounding areas. This distribution follows the geographic spread of MDR-TB in Peru for this period, where 87% of cases occur in the capital and its surrounding areas. Although the overall cure rate was significantly lower in XDR-TB than in MDR-TB, the timing of the DST result was significantly associated with an improvement in the treatment outcomes and the time to culture conversion. In fact, our study shows that ten of the fourteen XDR-TB cases (71%) with a DST result dated before or 31 days after starting treatment were cured under ITR. These results surpass those reported in Latvia where 61% of the XDR-TB cases experienced a favourable outcome, though less strict criteria were used to define XDR-TB (i.e. MDR-TB plus resistance to 3 or more of the 6 classes of second-line drugs) [2]. Our findings are in line with the outcome analysis of a group of XDR-TB cases from New York City [22] where XDR-TB diagnosed prior to starting treatment had better outcomes compared to those who acquired XDR-TB during treatment.

Looking at treatment history, 11.5% XDR-TB cases had never been treated with any anti-TB drugs and 40.5% had never been treated with second-line drugs. This information provides a rough estimate of the proportion of primary XDR-TB in Peru but without genetic confirmation, it is not possible to accurately estimate the proportion of cases who acquired XDR-TB during treatment. Nonetheless, this analysis for XDR-TB cases points out an important distinction. Under programmatic conditions, primary MDR-TB cases are defined as those that were diagnosed through a DST performed before beginning the very first anti-TB treatment (i.e. cases that were never treated before). In the context of XDR-TB however, the concept of primary resistance becomes more complex. While XDR-TB cases that were never treated before can be labelled as primary cases, we consider that monitoring the number of XDR-TB cases that have never been exposed to second-line drugs (particularly fluoroquinolones or second-line injectables) is equally important, and fit a more nuanced category of ‘probable primary’ resistance.

In our study, XDR-TB patients generally had less favourable outcomes and longer times to culture conversion than those with MDR-TB, which is evidence to the greater challenge of treating and controlling XDR-TB. However, a more careful evaluation suggests that XDR-TB cases can have similar outcomes to their MDR counterparts; when the DST was done close to treatment initiation, 71% of XDR-TB cases were cured, compared to 72% for MDR-TB patients. The results of the stratified analysis support the idea that having first- and second-line DST performed prior to treatment initiation can considerably improve the chances of cure, even for XDR-TB, provided the WHO treatment guidelines are followed [20] . Early DST can also result in quicker culture conversion, reducing the infectious period and disease transmission, even in the case of XDR-TB. DST results will in fact inform the composition of an ITR which, delivered under DOT, will allow use of effective drugs against the bacillus with consequent treatment of the disease and control of the amplification of resistance during treatment [23], [24].

Though the NTP suggests ending treatment at the 24th month, it is common that treatment be prolonged beyond this period, especially in patients that are persistently culture-positive. Consequently, programmatic outcomes (i.e. outcomes assigned by the CER and recorded in the registry) do not always match the outcomes following the consensus definitions [19]. In our study, this happened for 3 XDR-TB cases. All three failed by programmatic standards but did not have the culture information to assign a consensus definition. Disparities between programmatic and consensus definitions were greater in the MDR group where 87% of 30 cases labelled treatment completers were cured by programmatic standards. Hence, difference in cure rates for MDR-TB and XDR-TB would be more pronounced under programmatic standards.

A limitation of this study is the small sample size of the XDR-TB cohort, which affects the power of statistical tests, as well as the reliability of estimates of conversion time and cure rates. For example, although no significant difference was detected when comparing the proportion of cures of XDR-TB patients who underwent STR and ITR. However, due to the small sample size, the chances of detecting any difference if there was one, was estimated between13.9% and 15.2%. Similarly, the second strata of the XDR group showed no significant difference in the proportion of patients cured compared to the MDR counterparts, but the chance of detecting the difference given the sample size was approximately 20%. In both cases, the estimated power of the analysis was well below the usually recommended value of 80%.

The cohort analysis of XDR-TB patients reveals better treatment outcomes compared to previous reports [1], [3]. The striking difference in outcomes compared to the report from South Africa could be explained in part by the different levels of HIV co-infection. It is unknown whether treatment outcomes of XDR-TB treatment programmes in settings with relatively low HIV burden such as Peru, can be reproduced in areas with high HIV prevalence. However, our data show that in the absence of HIV, XDR-TB is a treatable disease.

In Peru, HIV prevalence is relatively low [12] and this is reflected in this study by the fact that no cases of XDR-TB/HIV were detected. However, two potential confounders could cause an underestimation of XDR-TB/HIV co-infection. First, the coverage of HIV testing among MDR-TB patients was extremely low before 2005, when policy was implemented recommending HIV testing for all patients approved for treatment with second-line drugs. Second, before March of 2005 patients had to fail STR before getting a second-line DST, a circumstance where XDR/HIV co-infected patients could have died before receiving second-line DST.

### Conclusion

Peru is a setting with a well-established TB control program, large experience treating MDR-TB and a low HIV burden. Given that the laboratory capacity of performing second-line DST has greatly increased recently, it is likely that the number of notified XDR-TB cases will also increase in the coming years. Nevertheless, this study presents evidence suggesting that in the Peruvian context, a large proportion of XDR-TB patients can be cured if DSTs for first and second-line drugs are performed early and treatment is delivered according to the WHO Guidelines. Though the overall cure rate was low compared to MDR-TB patients, outcomes and time to culture conversion were significantly better in patients who received DST close to treatment initiation when the ITR is first tailored. Though other important factors that influence the chances of being cured were not analyzed (e.g. specific drugs used in treatment regimens and pulmonary surgical intervention), our findings provide a potentially favourable perspective for patients affected by this form of tuberculosis. This study reinforces the importance to accelerate efforts to develop laboratory capacity for universal access to DST to both first- and second-line anti-TB drugs as well as to introduce rapid diagnostic tools for MDR and XDR-TB [25].

**Disclaimer:** MZ and EJ are staff members of the World Health Organization. The authors alone are responsible for the views expressed in this publication and they do not necessarily represent the decisions or policies of the World Health Organization.
